# Development of Ultrahigh Permeance Hollow Fiber Membranes via Simple Surface Coating for CO_2_/CH_4_ Separation

**DOI:** 10.3390/molecules27238381

**Published:** 2022-12-01

**Authors:** Noresah Said, Kar Chun Wong, Woei Jye Lau, Ying Siew Khoo, Yin Fong Yeong, Nur Hidayati Othman, Pei Sean Goh, Ahmad Fauzi Ismail

**Affiliations:** 1 Advanced Membrane Technology Research Centre (AMTEC), Faculty of Chemical and Energy Engineering, Universiti Teknologi Malaysia, Skudai 81310, Johor, Malaysia; 2 Department of Chemical Engineering, Universiti Teknologi PETRONAS, Bandar Seri Iskandar 32610, Perak, Malaysia; 3 Department of Oil and Gas Engineering, School of Chemical Engineering, College of Engineering, Universiti Teknologi MARA, Shah Alam 40450, Selangor, Malaysia

**Keywords:** coating, Pebax, silicon, hollow fiber membrane, high permeance, CO_2_ separation

## Abstract

Most researchers focused on developing highly selective membranes for CO_2_/CH_4_ separation, but their developed membranes often suffered from low permeance. In this present work, we aimed to develop an ultrahigh permeance membrane using a simple coating technique to overcome the trade-off between membrane permeance and selectivity. A commercial silicone membrane with superior permeance but low CO_2_/CH_4_ selectivity (in the range of 2–3) was selected as the host for surface modification. Our results revealed that out of the three silane agents tested, only tetraethyl orthosilicate (TEOS) improved the control membrane’s permeance and selectivity. This can be due to its short structural chain and better compatibility with the silicone substrate. Further investigation revealed that higher CO_2_ permeance and selectivity could be attained by coating the membrane with two layers of TEOS. The surface integrity of the TEOS-coated membrane was further improved when an additional polyether block amide (Pebax) layer was established atop the TEOS layer. This additional layer sealed the pin holes of the TEOS layer and enhanced the resultant membrane’s performance, achieving CO_2_/CH_4_ selectivity of ~19 at CO_2_ permeance of ~2.3 × 10^5^ barrer. This performance placed our developed membrane to surpass the 2008 Robeson Upper Boundary.

## 1. Introduction

Natural gas is a naturally occurring hydrocarbon gas combination that mostly consists of methane (CH_4_) and carbon dioxide (CO_2_). However, the composition of natural gas varies from one area to another, and its quality is greatly dependent on the number of impurities [1]. Since CO_2_ is very corrosive and can ruin pipes and equipment, removing it before delivering natural gas to a pipeline is required in most cases. Gas separation technology is important for these applications, and membrane-based technology, in particular, has received increasing attention over the past decade as it offers several advantages over conventional techniques, including lower energy consumption, simple process, compactness (reduced system footprint), free of chemical usage and scalability [1,2,3,4].

The development of highly gas-permeable membranes is critical for the industrial application of gas separation membranes [5,6]. Polymeric membranes have been extensively developed owing to their accessibility and low cost [5,7]. Both flat sheet and hollow-fiber membranes can be applied for CO_2_ separation but comparing these two types of membrane configurations, it can be stated that hollow fibers are easier to scale up, owing to their much higher packing density and self-supporting structure. Compared to the commonly used polymers such as polysulfone (PSf), polyethersulfone (PES), and polyvinylidene fluoride (PVDF) for the preparation of hollow fibers, silicone, e.g., polydimethylsiloxane (PDMS) is rarely used as material for membrane substrate even though it can offer very high permeance [7,8]. The main problem associated with the silicon-based membrane is its low separation efficiency for CO_2_/CH_4_ pair. In view of this, modifications of the surface of the silicone membrane are necessary in order to improve its selectivity while offering superior gas permeance.

Ideally, a high-performance membrane should exhibit both excellent permeability and selectivity. Most importantly, it should be able to surpass the Robeson limit. Studies have been previously carried out to investigate the effects of surface coating on the membrane surface properties, as coating is the simplest approach to optimizing the membrane gas separation efficiency. Madaeni et al. [8] investigated the effect of the dip-coating method on the performance of PES support with respect to CO_2_ permeability and selectivity. In the dip-coating method, support was immersed in a coating solution of different PDMS concentrations for 3 min. The authors reported that the surface defects of the support could be minimized upon coating. However, the coating layer adversely affected the CO_2_ permeability of the membrane. Upon five layers of PDMS surface coating, the results showed that the membrane selectivity was not significantly improved owing to the penetration of PDMS coating solution into the pores of PES membranes.

On the other hand, Abdul Karim et al. [9] developed a PDMS-metal organic framework hybrid layer on the PSf membrane and found that the presence of the layer played an important role in improving gas permeance and selectivity which contributed to higher affinity towards CO_2_ due to the presence of highly CO_2_-adsorptive filler within the coating layer. Roslan et al. [10] improved the surface properties of PSf hollow fiber membrane for CO_2_/CH_4_ separation by forming a selective layer composed of polyether block amide (Pebax) and graphene oxide (GO) atop the PDMS-coated membrane. The authors reported a lower degree of plasticization and very stable performance for the developed membrane over the 50-h operation, recording CO_2_/CH_4_ selectivity of 52.57 and CO_2_ permeance of 28.08 GPU.

Similarly, Moradi et al. [11] produced a selective PDMS layer on a thin film composite (TFC) membrane via the dip-coating method by immersing the membrane in the PDMS solution of different concentrations for a specific duration. The PDMS, which acted as a thin top layer, provided desired permeability and selectivity for gases by sealing the membrane surface pinholes. The selectivity for prepared PDMS-coated TFC membranes was in the range of 6.7–22.5. Using the dip-coating method, Suleman et al. [12] developed a PDMS-coated PSf membrane for CO_2_/CH_4_ separation. The PDMS coating solution was prepared by dissolving 20 wt% PDMS in n-hexane. The coated membranes were subjected to 30-min thermal treatment at 120 °C. The authors found that the swelling degree of the PSf membrane could be substantially reduced by having a PDMS layer atop the membrane. The best performing PDMS-coated membrane could achieve CO_2_/CH_4_ selectivity of 85 at an operating pressure of 10 bar.

In order to improve the membrane surface properties, some researchers introduced nanomaterials into the membrane matrix. Kiertalab et al. [13], for instance, incorporated GO nanoparticles into polyvinyl alcohol (PVA) modified Pebax matrix to prepare Pebax/PVA/GO ternary mixed matrix membranes (MMMs) for CO_2_/CH_4_ separation. The inclusion of GO was reported to improve CO_2_ permeability and showed more efficient performance in CO_2_/CH_4_ separation. This is mainly because the presence of GO within the Pebax matrix developed an extra intermolecular space which enhanced the CO_2_ permeability through the MMMs. Sustrisna et al. [14] also utilized nanofillers to improve the selective layer of the membrane. Firstly, the researchers formed a PDMS gutter layer on the PVDF hollow fiber membrane. It was followed by selectivity layer coating using a solution composed of 3 wt% Pebax and 0–30 wt% ZIF-8. The incorporation of ZIF-8 nanoparticles was found to play a positive role in improving the gas permeability with only a marginal loss of selectivity. The stable structure of ZIF-8 and the hydrogen bonds between ZIF-8 organic ligands significantly improved the linear glass polymer chain stiffness, ensuring the good operational stability of the membrane under elevated pressure and hollow fiber membranes.

From these studies, we found that although the researchers reported improved membrane gas separation performance (in particular CO_2_/CH_4_ selectivities) upon surface coating/modification, some of the developed membranes suffered from permeance reduction (some even lower compared to the unmodified membranes) [15,16,17]. Furthermore, although the incorporation of nanoparticles into the coating layer could improve membrane gas separation efficiency, large-scale fabrication of these membranes might not be practical and economical as the use of advanced nanoparticles is always associated with high manufacturing costs, and its long-term stability in the coating layer remains largely uncertain.

In view of this, the primary objective of this work is to develop a multilayer hollow fiber membrane using a simple coating technique and coating materials that can surpass the 2008 Robeson Upper Boundary for CO_2_/CH_4_ separation. To achieve this target, we first selected a commercially available silicone hollow fiber membrane that displays superior permeance for CO_2_ and CH_4_ (but low CO_2_/CH_4_ selectivity in the range of 2–3). The base membrane was then subjected to a series of surface modifications, including different types of silane agent coating, different layers of silane agent coating, and Pebax coating (to further seal any surface defects on the membrane). The changes in the membrane surface properties were analyzed using a Fourier-transform infrared (FTIR) spectroscope and field emission scanning electron microscope (FESEM), while the separation performance of the membranes was evaluated with respect to permeance and CO_2_/CH_4_ selectivity using a lab-scale gas permeation system. At last, we compared the CO_2_/CH_4_ selectivity of our developed membranes with the Robeson Upper Boundary as a function of CO_2_ permeability.

## 2. Results and Discussion

### 2.1. Effects of Silane Coating on Membrane Properties

**Figure 1:**
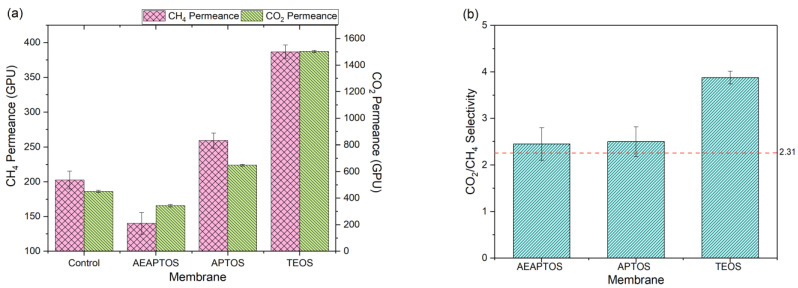
(**a**) CH_4_ dan CO_2_ gas permeance of control silicone membrane and different silane-coated membranes tested at 3 bar and (**b**) CO_2_/CH_4_ gas pair selectivity of different silane-coated membranes (Note: The red dotted line indicates the selectivity of control membrane).

Figure 1 presents the performance of silicone hollow fiber membranes with and without coating on their outer surface with respect to gas permeance and CO_2_/CH_4_ gas pair selectivity. As can be seen, although the control hollow fiber membrane exhibits extremely high permeance for CO_2_ and CH_4_, it does not offer promising CO_2_/CH_4_ selectivity. Among the silane-coated membranes, it is found that the TEOS-coated membrane demonstrated the highest CO_2_/CH_4_ gas pair selectivity of 3.49. The AEAPTOS and APTOS, meanwhile, only show a marginal improvement in the gas pair selectivity of 2.45 and 2.47, respectively. In terms of permeance, the AEAPTOS-coated membrane exhibits an even lower value than the control membrane. Surprisingly, the AEAPTOS and APTOS, which contain an amine group in their structure, resulting in lower membrane selectivity (upon coating) compared to the TEOS. Usually, polymers with amine groups show higher CO_2_ solubility, leading to higher selectivity [18,19]. There are several reasons causing the contradictory results. First, the presence of amine groups in the AEAPTOS and APTOS might have a lower degree of compatibility with the silicon membrane, which affects their positive features in improving membrane selectivity. Second, both AEAPTOS and APTOS, which have longer chains than TEOS, might not be able to form a denser and thinner structure compared to the TEOS coating. We ruled out the impact of ethanol coating solution on the structure of the membrane as the coating duration is short (5 min), and silicon has reasonably good chemical resistance against ethanol.

**Figure 2:**
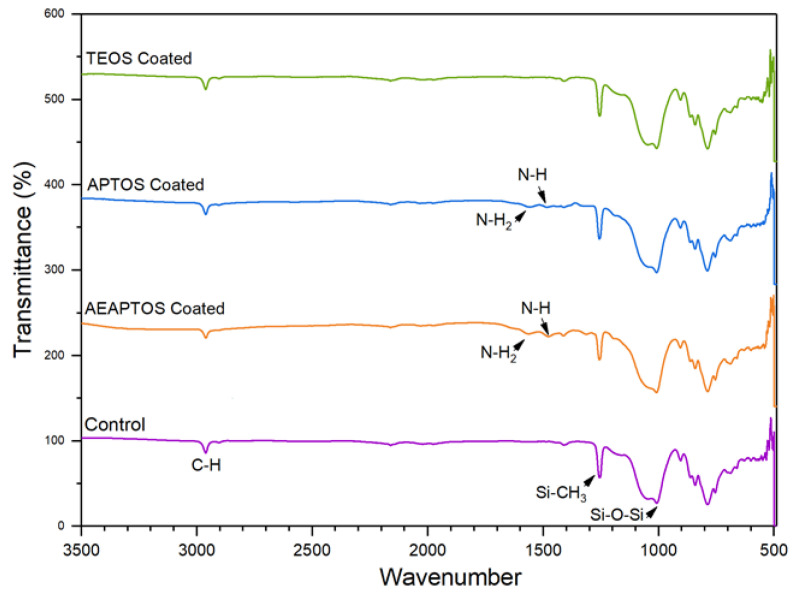
FTIR silicone hollow fiber membrane with and without surface coating.

Figure 2 compares the FTIR spectra of silicone membranes coated with different silane agents. All of the membranes show very similar peaks at 2950, 1240, and 1000 cm^−1^. The symmetrical vibration of Si-CH_3_ at 1250 cm^−1^ is attributed to the organic structure of the silicone [20,21] membrane, which is used as a substrate for all membranes. The peaks at 2950 and 1000 cm^−1^ corresponded to the C-H stretching and Si-O-Si group of both silicone membrane and the coating materials [21,22]. For the AEAPTOS and APTOS-coated membranes, additional peaks at 1590 cm^−1^ (N-H_2_) and 1490 cm^−1^ (N-H) are detected. The presence of these two peaks confirms the successful coating of the silicone membranes.

Considering that the TEOS-coated membrane offers higher permeance and selectivity compared to the other two coated membranes, further investigations are performed to examine if multilayer TEOS coating could lead to greater gas pair selectivity while offering good permeance compared to the membranes reported in the literature.

### 2.2. Effects of TEOS Coating Layers on Membrane Properties

**Figure 3:**
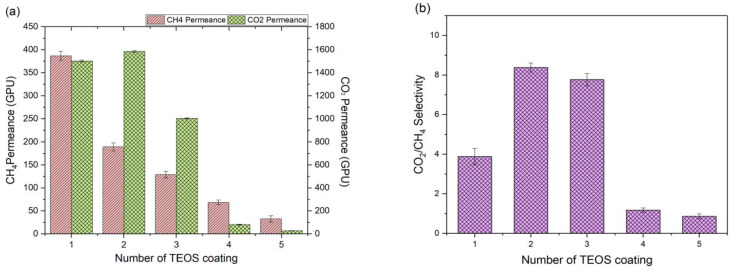
The impact of the TEOS coating layer on the hollow fiber membrane with respect to (**a**) CH_4_ and CO_2_ gas permeance measured at 3 bar and (**b**) CO_2_/CH_4_ gas pair selectivity.

**Figure 4:**
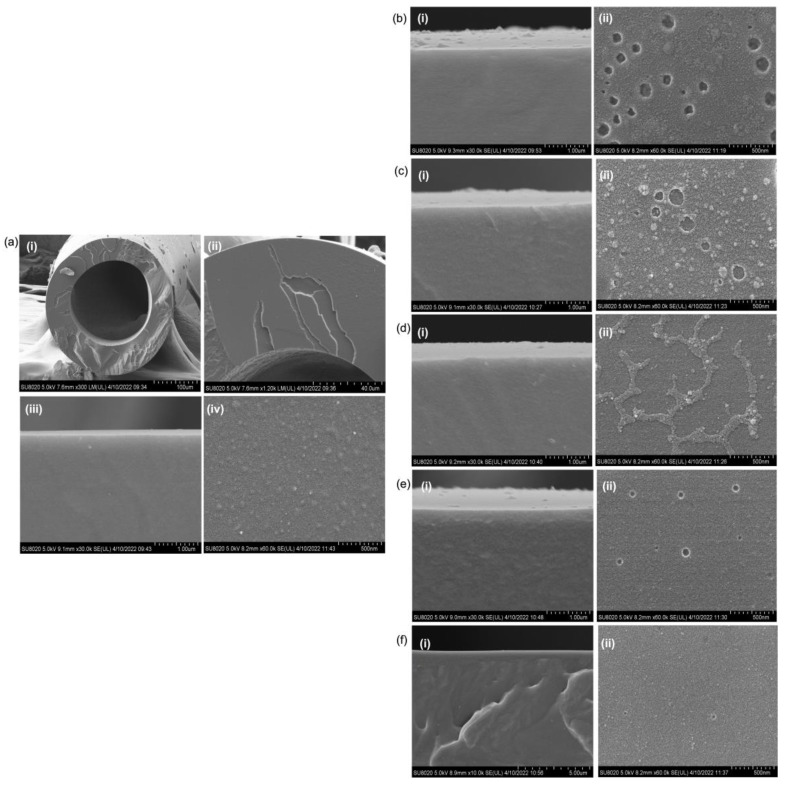
(**a**) FESEM images of silicone membrane, (**i**,**ii**) full and partial cross-sectional structure, and (**iii**,**iv**) cross-section near the outer surface and outer surface. Impact of TEOS coating layers on the membrane cross-section (**i**) and surface (**ii**), (**b**) single layer, (**c**) two layers, (**d**) three layers, (**e**) four layers, and (**f**) five layers.

The impacts of multilayer TEOS coating on the surface of silicone hollow fiber membrane were studied at 3 bar and room temperature, and the results are shown in Figure 3a,b. Figure 4a shows the cross-sectional and surface morphology of the control silicone hollow fiber membrane. The inner and outer diameters of the fiber are very close to the manufacturer’s data, i.e., 190 and 300 µm, respectively. By increasing the number of TEOS layers from 1 to 5, the CH_4_ permeance of the membrane is found to decrease accordingly. This is mainly due to an increase in the transport resistance of CH_4_ as a result of the increased thickness of the coating layer. Similarly, CO_2_ permeance is also found to decrease sharply by increasing the coating layers. Apart from the contribution of increasing thickness, the sealing effect imposed by subsequent TEOS layers on existing coating is also possible. This can be visibly observed from FESEM images whereby the void-riched structure of a single TEOS layer (Figure 4b) transited into a more compact structure with higher coating cycles as the TEOS solution penetrated the existing coating layer to fill up the voids and sealed them upon drying.

By comparing the membrane coated with 2 and 3 TEOS layers, it is found that the two layers of coating are better at achieving higher CO_2_/CH_4_ selectivity with better gas permeance. The surface pin holes are greatly reduced upon the two-layer coating, which leads to an improvement in gas separation (Figure 4c). Although the surface pin holes are absent when the membrane is coated with 3 TEOS layers, several strands are developed on the coated membrane surface (Figure 4d). Such morphology further increases the membrane gas transport resistance, which results in lower gas permeance.

By further increasing the TEOS layers from 3 to 4 and 5, membrane selectivity plummeted to 1.17 and 0.86, respectively. Such a phenomenon can be caused by surface cracking (due to the formation of a very thick coating layer) which does not offer a good feature to separate gas molecules. From the cross-sectional image (Figure 4e), the coating layer contains a significant number of voids which is the main reason why this membrane has poor gas separation efficiency. Similar voids are also detected in the membrane coated with a single layer of TEOS, and this membrane also suffers from low CO_2_/CH_4_ selectivity. At the highest number of coating layers, the coating layer could not be clearly seen (Figure 4f), and this finding strongly suggests the possible crack of the coating layer, which makes it unable to adhere firmly to the fiber. The TEOS coating layer might delaminate entirely from the substrate surface due to extensive defects or cracks, which led the overly thick layer to fail in supporting its own mass.

Given this, the membrane coated with 2 TEOS layers is more feasible for further investigation by considering its good performance between permeance (compared to 3 coating layers) and CO_2_/CH_4_ selectivity (compared to other coated membranes). In terms of surface chemistry, there is no obvious difference between the peaks of the membranes measured by FTIR (see Figure S1), except for the peak at 1250 cm^−1^, where the single-layer coated membrane shows higher intensity than other coated membranes. The peak is attributed to the Si-C vibration of the silicone material [11,23], and the reduction in the peak intensity with an increasing number of coating layers is due to an increase in the thickness of the coating layer on the silicone membrane surface.

### 2.3. Effects of Pebax Coating on TEOS-Modified Membrane

**Figure 5:**
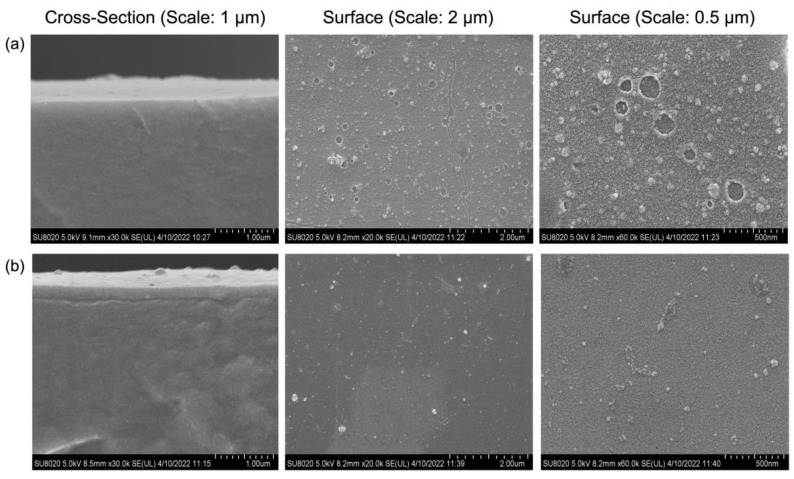
FESEM cross-section and surface images of TEOS-coated membrane (2 coating layers of TEOS), (**a**) without Pebax layer and (**b**) with Pebax layer.

In order to seal the surface defects of the TEOS-coated membrane, we employed Pebax as an additional coating layer to improve the properties of the TEOS-coated membrane. From Figure 5, it can be seen that the voids present on the surface of the TEOS-coated membrane are effectively sealed by the Pebax layer, which produces a much smoother surface morphology.

**Figure 6:**
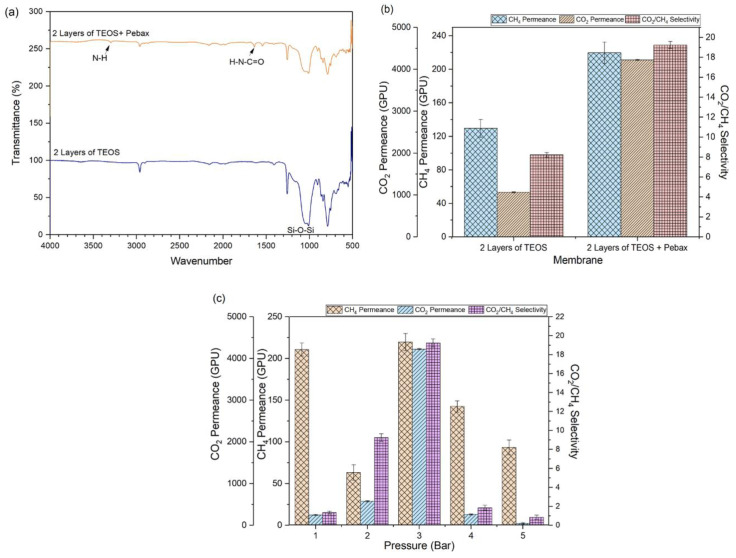
(**a**) FTIR spectra of two different coated membranes, (**b**) Gas permeance and selectivity of TEOS-coated membranes with and without additional Pebax layer tested at 3 bar, and (**c**) impact of operating pressure on TEOS/Pebax coated membrane on gas separation performance.

Figure 6a further confirms the presence of a Pebax layer on the resultant membrane surface owing to the detection of two specific functional groups of Pebax, i.e., N-H (3200 cm^−1^) and H-N-C=O (1650 cm^−1^). Figure 6b meanwhile compares the performance of the TEOS-coated membrane with and without the Pebax layer. As shown, the selectivity of the membrane is increased from 8.23 to 19.24 upon the Pebax coating, recording ~130% enhancement in separation. These results are due to the intrinsic characteristics of Pebax, which show exceptionally large solubility coefficients against CO_2_. The existence of polar groups (ether oxygen atoms) in the polymer backbone is the key factor that enhances the selectivity for CO_2_ [24,25].

The effect of operating pressure on the performance of TEOS/Pebax coated membrane was also studied, and the results are presented in Figure 6c. The results clearly show that increasing operating pressure from 3 to 4 and 5 bar does not help in improving membrane permeance and selectivity. The membrane demonstrates extremely low CO_2_/CH_4_ selectivity (<2) when tested at 4 and 5 bar. Our findings are consistent with the manufacturer’s datasheet in which the maximum operating pressure for silicone hollow fiber membrane should be set at 3 bar. The decrease of CH_4_ permeance from 1 to 2 bar is mainly due to the compression of the membrane. Since the mechanism governing CH_4_ permeation is diffusion (physical) while CO_2_ is both diffusion (physical) and solubilization (chemical interaction with polymeric chain), thus compression of the membrane will have a greater impact on CH_4_ than CO_2_. When the pressure is further increased from 2 bar to 3 bar, the silicone-based membrane’s integrity starts to be affected, i.e., it begins to stretch and develop the pinhole. At this stage, the permeance of both gases is greatly improved because the resistance from the support layer is minimal compared to the testing conditions at only 1 or 2 bar. However, this pressure is not enough to destroy the dense selective layer, and the membrane still achieves improved selectivity.

Typically, the silicone-based membrane has very poor resistance against high operating pressure. This rubbery membrane has a highly flexible network that is prone to deformation when subjected to high pressure [26]. When excessive pressure is applied (i.e., >3 bar in this study), severe membrane deformation causes its structure to collapse. The collapse of the hollow fiber membrane would then constrict the membrane lumen, limiting the passage of gas from the membrane into the bubbling soup setup and resulting in low permeation reading. Since lumen constriction was more severe at higher pressure, the sample exhibited lower permeance at 5 bar than at 4 bar. Moreover, the collapse of membrane structure is often accompanied by the formation of defects, such as cracks or tears, which compromise the selectivity of the membrane.

**Figure 7:**
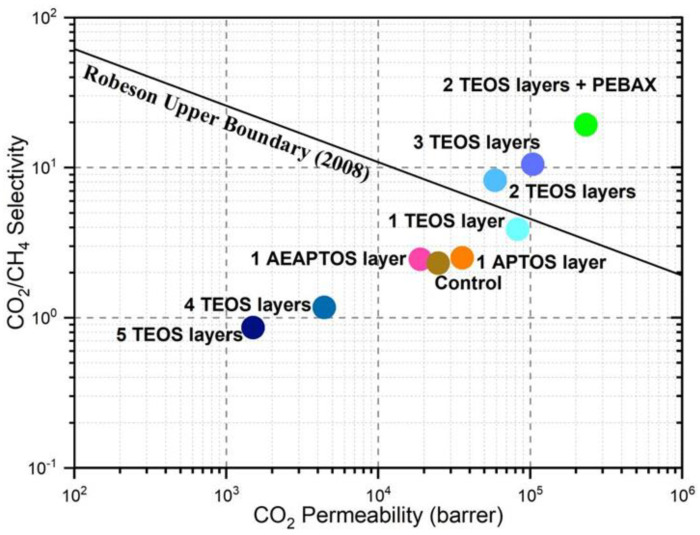
Performance comparison of different coated membranes synthesized in this work with the Robeson Upper Boundary.

Figure 7 compares the CO_2_/CH_4_ selectivity of our modified membranes with the Robeson Upper Boundary as a function of CO_2_ permeability. Of the membranes included in this graph, three membranes, i.e., the ones coated with 2 TEOS layers followed by Pebax, 2 TEOS layers, and 3 TEOS layers, are located above the boundary while the rest (6 membrane types) appear lower than the boundary. Although the control silicone membrane has high CO_2_ permeability, its CO_2_/CH_4_ selectivity is quite low (~2.31). Our approach indicates that by forming suitable layers of TEOS on the control membrane followed by Pebax coating, the intrinsic separation properties of the silicon membrane can be enhanced. Our best-performing membrane (i.e., 2 TEOS layers + Pebax) could achieve much higher CO_2_/CH_4_ selectivity (~19) while demonstrating superior CO_2_ permeability at 2.3 × 10^5^ barrer than the control membrane. These results indicate that the developed membrane is eight times more selective and nine times more permeable than the control membrane.

Table 1 compares the results of our best performing membrane with other surface-modified membranes reported in the literature. As can be seen, although our membrane demonstrates relatively low CO_2_/CH_4_ selectivity, its CO_2_ permeance is the highest owing to the use of silicone membrane with high permeance characteristics as support for coating. Nevertheless, our membrane is unsuitable for operating at pressure >3 bar due to this low resistance against compaction. Compared to other research studies that utilized expensive nanomaterials such as metal-organic framework [9] and GO [10] to improve membrane gas performance via the surface coating method, our approach seems more practical and economical for membrane manufacturing.

## 3. Materials and Methods

### 3.1. Chemicals and Materials

The silicone hollow fiber substrate (PDMS, (C_2_H_6_OSi)_n_) used in this work was supplied by PermSelect (Ann Arbor, MI, USA) and had an outer diameter (OD) and inner diameter (ID) of 190 µm and 300 µm, respectively. The fibers made of polydimethylsiloxane (PDMS, (C_2_H_6_OSi)_n_) were supplied in a small module (PDMSXA-7500) with a total effective surface area of 7500 cm^2^. Based on the manufacturer’s datasheet, this silicone hollow fiber substrate could achieve a CO_2_/CO_4_ separation factor of 3 when tested with CO_2_/CH_4_ mixed gas (50/50 in *v*/*v*). To modify the outer surface of hollow fiber substrate with silane agents, N-(2-aminoethyl)-3-aminopropyltrimethoxysilane (AEAPTOS), (3-aminopropyl)triethoxysilane (APTOS) and tetraethyl orthosilicate (TEOS) obtained from Sigma Aldrich were respectively used.

**Figure 8:**
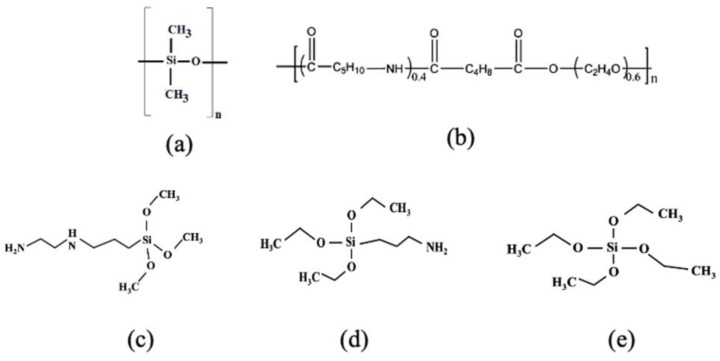
Organic structure of (**a**) PDMS, (**b**) Pebax, (**c**) AEAPTOS (MW: 222.36 g/mol), (**d**) APTOS (MW: 221.37 g/mol) and (**e**) TEOS (MW: 208.33 g/gmol).

Separately, polyether-blockamide (Pebax, MH1657) obtained from Arkema (King of Prussia, PA, USA) was also used as a coating material to improve the surface properties of the silicone hollow fiber substrate. Analytical-grade ethanol purchased from Sigma Aldrich was used during the membrane surface coating process. For membrane gas performance evaluation, pure CO_2_ and CH_4_ gases (>99.99%) were supplied by Alpha Gas Solution Sdn. Bhd. (Shah Alam, Malaysia) were used. Lastly, epoxy adhesive resin (Loctite, Dusseldorf, Germany) was used to prepare the lab-scale membrane module for in-house evaluation. The organic structures of PDMS and different coating materials are presented in Figure 8.

### 3.2. Membrane Surface Coating

#### 3.2.1. Single Layer Coating

In this work, the outer surface of the hollow fiber substrate was modified using different silane agents. Prior to surface coating, three different types of silane coating solutions (i.e., AEAPTOS, APTOS, and TEOS) were first prepared. For each solution, 2 wt% silane agent was dissolved in ethanol and stirred overnight to obtain a homogeneous phase. As the first step of the experiment, the hollow fiber substrate (with a length of 3–5 cm) was subject to single coating by immersing it in the coating solution and remained for 5 min before taking it out. One side of the hollow fiber substrate was sealed with epoxy resin to prevent the coating solution from penetrating through the lumen of the fiber. Upon completion of the coating process, the respective coated membrane was dried naturally for 5 h at room conditions.

#### 3.2.2. Multilayer Coating

For the multilayer coating process, only the best silane coating solution was selected by identifying the single layer-coated membrane that showed the highest CO_2_/CH_4_ selectivity and promising gas permeance. In this work, the TEOS coating solution was chosen. Similar to the single-layer coating approach, the multi-layer coating was carried out repeatedly on the same substrate. The number of coatings was limited to 5 as excessive coating could adversely affect the resultant membrane’s performance.

#### 3.2.3. Pebax Coating

The best performing multilayered membrane was further modified by subjecting it to additional Pebax coating to seal its surface defects for gas pair selectivity improvement. In brief, 2 wt% Pebax was dissolved in 70:30 ethanol: water at 70 °C followed by 24 h stirring in order to produce a homogeneous coating solution. After that, the multilayered membrane was immersed in Pebax solution and kept for 10 min before taking it out.

### 3.3. Membrane Gas Separation Performance Evaluation

The membrane modules were prepared by potting 2–3 hollow fibers with a length of no more than 5 cm. An epoxy adhesive resin was used as the potting resin. Upon completion of potting, the membrane modules were left to cure at room conditions for at least 24 h prior to gas permeation testing. After that, the membranes with and without surface coating were evaluated for gas permeance and pair selectivity.

**Figure 9:**
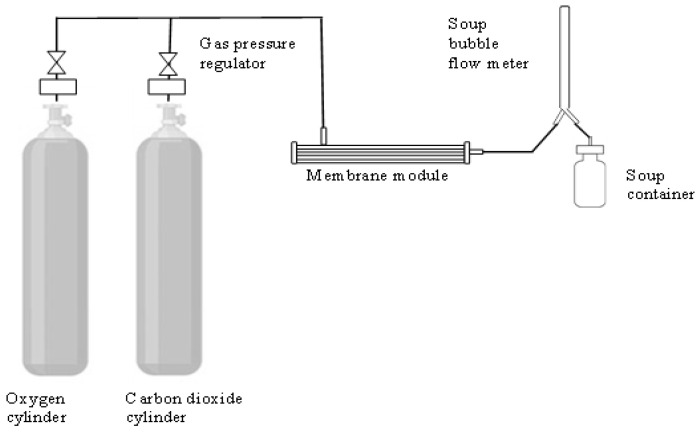
Schematic of gas permeation system for evaluating performance of different types of membranes.

The self-customized gas permeation system illustrated in Figure 9 was used to determine the membrane performance in gas permeance and gas pair selectivity. Two pure gases, i.e., CO_2_ and CH_4_, were utilized to access the membrane separation performance. Prior to the evaluation, the membrane module was inserted into a stainless-steel housing, followed by tightening it. The flow configuration used in this study was shell-side feed, i.e., the feed gas diffused from the gas cylinder through the membrane’s outer surface, and the permeated gas was collected from the fiber lumen. The permeated gas was then delivered to a soap bubble flow meter in which the gas permeance, *P_A_*/*I* (GPU, Note: 1 GPU = 10^−6^ cm^3^ (STP)/cm^2^·cm·Hg), was determined using the following equation.

(1) $$\frac{P_{A}}{I}=\frac{273.15\times 10^{6}Q}{A\times \Delta P\times T}$$

where *Q* is the volumetric flow rate of gas diffusing across the membrane (cm^3^/s at STP), *A* is effective membrane area (cm^2^), ΔP is transmembrane pressure (cm·Hg), and *T* is operating temperature (K). The experiments were conducted at room temperature (24 °C ± 1) with pressure ranging from 1 to 5 bar. For each membrane sample, the experiments were repeated at least five times to yield average results. The membrane selectivity, $\propto_{A/B}\;$describing the ratio of gas pair permeability, was then calculated by the pressure-normalized flux of CO_2_ (A) over CH_4_ (B).

(2) $$\propto_{A/B}=\frac{P_{{\mathrm{CO}}_{2}}}{P_{{CH}_{4}}}$$

### 3.4. Membrane Characterization

Attenuated total-reflectance Fourier-transform infrared (ATR-FTIR) spectroscope (PerkinElmer Inc, Waltham, MA, USA) was employed to study the surface chemistry of the membrane before and after coating. Each analysis was performed on a dried membrane sample, and the scanning range was set between 500 and 4000 cm^−1^ with 32 scans at a rate of 4 cm^−1^. A field emission scanning electron microscope (FESEM, Hitachi, Japan) was used to compare the structural morphologies of hollow fiber membranes modified by different materials. The fiber was gently fractured in liquid nitrogen to produce a clean and smooth cross-section before transferring it to a metal plate with carbon tape on the lateral edges. For the membrane surface analysis, a short fiber (~0.5 cm) was placed horizontally on the metal plate. Before being analyzed, the membrane samples were sputter-coated with gold. The cross-sections and outer surfaces of the membranes were then examined using FESEM at different magnifications.

## 4. Conclusions

Although silicone membranes could achieve high permeance for gas molecules, their low CO_2_/CH_4_ selectivity (in the range of 2–3) makes them unsuitable for application. In the present work, we adopted a simple coating technique to improve the properties of silicone hollow fiber membranes using two different kinds of materials, i.e., silane agent and Pebax. Our results indicated that out of the three silane agents tested (AEAPTOS, APTOS, and TEOS), only TEOS improved the control membrane’s permeance and selectivity. Further investigation revealed that higher CO_2_ permeance and selectivity could be attained by coating the membrane surface with two layers of TEOS. This greatly improved CO_2_/CH_4_ selectivity from ~4 in the single-layer coated membrane to >8. From the FESEM images, we found that the surface integrity of the TEOS-coated membrane was greatly improved upon the coating of an additional Pebax layer. This additional layer sealed the pin holes of the TEOS layer and enhanced the resultant membrane’s selectivity. Our best-modified membrane could achieve CO_2_/CH_4_ selectivity of ~19 while demonstrating superior CO_2_ permeability at 2.3 × 10^5^ barrer. This placed our developed membrane above the Robeson Upper Boundary. Further study is also needed to examine if we can further improve the membrane performance by reversing the coating step on the membrane surface, i.e., Pebax coating followed by TEOS coating, to explore the full potential of TEOS as the uppermost membrane layer for gas separation.

## Figures and Tables

**Table 1:** Comparison of surface-modified hollow fiber membranes for gas separation with respect to CO_2_ permeance and CO_2_/CH_4_ selectivity.

Membrane	Coating Material	Operating Pressure (Bar)	CO_2_ Permeance (GPU or Barrer)	CO_2_/CH_4_ Selectivity	Year [Ref.]
Co-polyimide/graphene	Graphene/Pebax	6	3.3 GPU	28.6	2022 [27]
Polysulfone	PDMS/Pebax	5	39.56 GPU	34.28	2020 [28]
Polysulfone	PDMS/Pebax/Graphene oxide	5	28.08 GPU	52.57	2020 [10]
Polyetherimide	PDMS	2	190 GPU	~9.8	2021 [29]
Polyvinylidene fluoride	ZIF-8/Pebax	2	~350 GPU	~12	2017 [14]
^a^ Mixed matrix membrane	-	2	1042 barrer	25	2021 [30]
Silicone	TEOS/Pebax	3	4150 GPU or 2.3 × 10^5^ barrer	~19	Our Work

^a^ This membrane is made of 6FDA-durene polymer and MFU-4 metal organic framework.

## Data Availability

Data will be made available on request.

## Supplementary Materials

The following supporting information can be downloaded at: https://www.mdpi.com/article/10.3390/molecules27238381/s1, Figure S1: Surface chemistry of different TEOS-coated membranes determined by FTIR.
