# Supplementary material for: Development of Ultrahigh Permeance Hollow Fiber Membranes via Simple Surface Coating for CO2/CH4 Separation
Source: Molecules. 2022 Dec 1;27(23):8381. doi: 10.3390/molecules27238381 (PMC9738885; doi:10.3390/molecules27238381)
Supplement: Supplementary file 1 [file molecules-27-08381-s001.zip › molecules-2047529-supplementary.pdf]

Supplementary Figure

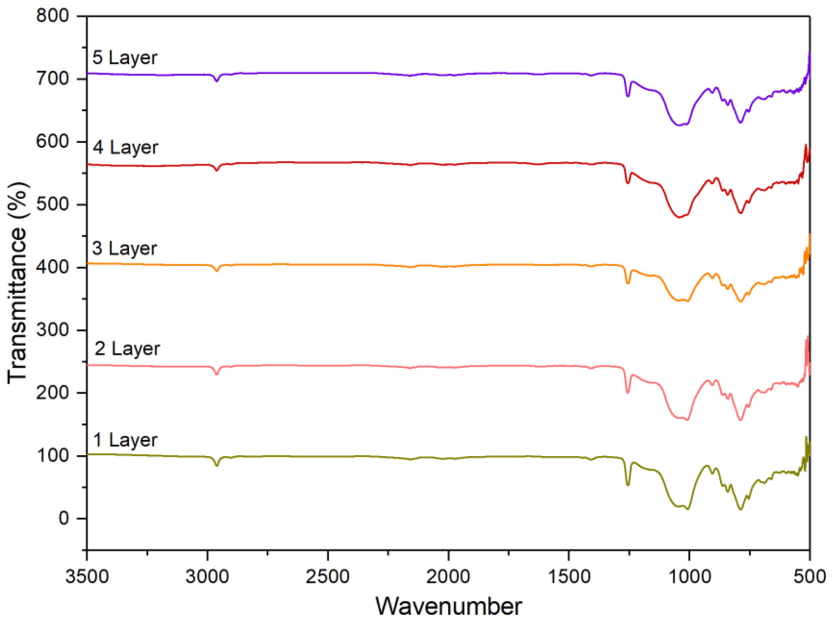

Figure S1. Surface chemistry of different TEOS-coated membranes determined by FTIR
